# One Small Step for a Patient, One Giant Leap for Orthostatic Hypotension

**DOI:** 10.7759/cureus.31612

**Published:** 2022-11-17

**Authors:** Amanda C Coniglio, Veraprapas Kittipibul, Ralph Pelligra, Eric S Richardson, Christopher L Holley, Marat Fudim

**Affiliations:** 1 Department of Medicine, Duke University School of Medicine, Durham, USA; 2 Department of Research, Ames Research Center, Mountain View, USA; 3 Department of Biomedical Engineering, Duke University, Durham, USA; 4 Department of Cardiology, Duke Clinical Research Institute, Durham, USA

**Keywords:** compression garment, heart failure, splanchnic compartment, autonomic dysfunction, orthostatic hypotension

## Abstract

A 52-year-old man with ischemic cardiomyopathy presented with progressive, severe orthostatic hypotension refractory to medical therapy. Standard abdominal and leg compression devices were used without success. A novel, inflatable abdominal compression device was created that alleviated the patient's symptoms and maintained his blood pressure.

## Introduction

Orthostatic hypotension (OH) is one of the most common causes of syncope (9%), being diagnosed in about 20-30% of people aged 65 years and older [1]. It is highly prevalent in patients with heart failure (HF) who have a high burden of comorbidities contributing to OH, as well as frequent use of neurohormonal and volume-modulating medications. The incidence of OH in patients with HF varies widely between 8 and 83%, with the large range in prevalence due to differences in age group and clinical setting being studied (inpatient versus outpatient) [2]. Many of the medications used to treat HF decrease blood pressure and blunt the body's compensatory responses, making OH a particularly challenging problem in HF patients. Here, we present a case of a profound case of OH in a patient with advanced HF in which an inflatable belt was developed to help alleviate a drop in blood pressure and restore quality of life. 

## Case presentation

A 52-year-old man (5'6" tall, 92.3 kg, BMI 32.8 kg/m2) with advanced ischemic cardiomyopathy (ejection fraction 20%) on home dobutamine presented to the emergency room with lightheadedness with standing. His other medical history included a left ventricular thrombus with prior strokes on warfarin, coronary artery disease with previous bypass graft surgery, and diabetes. His medications on admission included midodrine 5 mg three times a day, dobutamine 2.5 mcg/kg/min, spironolactone 25 mg twice a day, and empagliflozin 10 mg daily. His blood pressure was 141/83 mmHg with a heart rate of 81 bpm while lying down and 98/69 mmHg with a heart rate of 85 bpm with standing, reproducible on multiple occasions. His review of systems was otherwise negative.

An echocardiogram showed an ejection fraction of 20%, normal right ventricular function, and trivial valvular disease. A left heart catheterization showed patent grafts and stable native disease. A right heart catheterization on dobutamine 2.5 mcg/kg/min revealed a right atrial pressure of 7 mmHg, mean pulmonary artery pressure of 30 mmHg, a pulmonary capillary wedge pressure of 17 mmHg and a cardiac index of 1.7 L/min/m2 which was similar to his prior testing. His laboratory testing was notable for a hemoglobin A1c of 6.5%, normal renal function, thyroid studies, cortisol stimulation testing, and immunoglobulin-free light chains. CT brain imaging revealed stable, remote infarctions in the left middle cerebral artery distribution without any new infarcts or lesions. He underwent a tilt table test that was consistent with severe orthostatic hypotension (OH) (Table 1).

**Table 1:** Tilt table results The test was completed while the patient was on dopamine 5mcg/kg/min and taking midodrine 20mg three times a day, fludrocortisone and pyridostigmine

Duration (minutes)	Heart rate (beats per minute)	Systolic blood pressure (mmHg)	Diastolic blood pressure (mmHg)	Comments
0	72	120	85	Tilt table started
1	78	87	57	
3	86	81	52	Blurred vision 3/10
5	88	63	51	Blurred vision 5/10
7	90	62	52	Blurred vision 7/10, lightheaded 8/10, weak legs
9	97	71	42	Blurred vision 7/10, nausea 8/10, weak legs
10	78	76	42	Patient vomiting
12	118	118	79	Blurred vision 4/10, tilt ended

**Figure 1:**
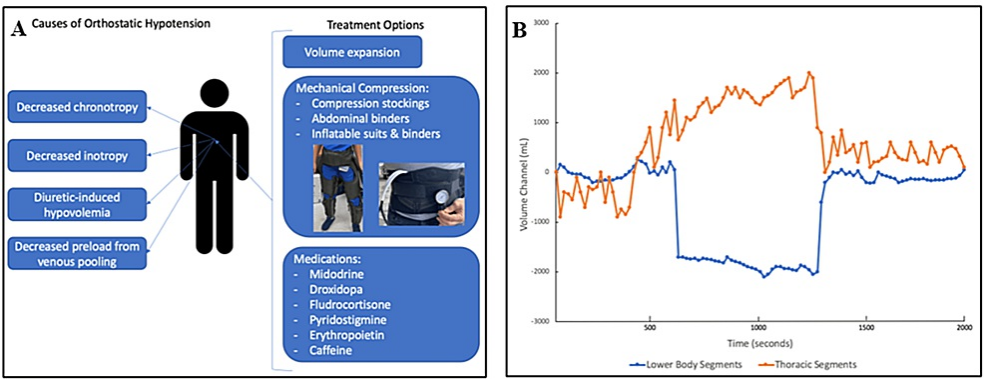
Mechanisms of orthostatic hypotension and non-inflatable antishock garment A. Mechanisms and treatment options for orthostatic hypotension in patients with heart failure. B. Fluid translocation with the non-inflatable antishock garment in a healthy adult male. At 10 minutes (600 seconds) an antishock compression garment was applied to the lower extremities of the subject and remained in place for 10 minutes total until its removal (removed at 20 minutes into the study period). Bioimpedance was used to evaluate segmental (thorax/abdomen) shifts in body fluid throughout the study period showing a shift in fluid from the lower body segments to the thoracic cavity when the antishock garment was applied. The use of the inverse proportional relationship of volume to the respective electrical resistance (Vgeom~1/R) and the effective resistant coefficient allows computing the volume of the respective segment. Original figure created by authors

His dobutamine was switched to dopamine 5 mcg/kg/min, midodrine was increased to 20 mg three times a day, and he was started sequentially on fludrocortisone, pyridostigmine, and droxidopa, which were all titrated to maximal doses. Despite a combination of an off-the-shelf surgical abdominal binder, compression stockings, and maximal medical therapy, the patient continued to be bed-bound for weeks due to profound OH. Given the significance of abdominal venous pooling in the pathophysiology of OH, the decision was made to try to provide a higher level of compressive therapy, which could not be achieved with available compression garments. The medical team contacted NASA to obtain a compression suit (G-suit) and in parallel developed an inflatable abdominal belt using Duke Biomedical Engineering resources (see Figure 1), which allowed for manual adjustment of the cuff pressure. The patient tolerated a pressure around 50 mmHg and, with its use alone, was able to tolerate a standing position with a systolic blood pressure drop of only ~10 mmHg without symptoms. Starting the first day of belt application, the patient was able to ambulate. In the subsequent week, the patient was weaned off all oral OH therapies, including the dopamine drip. The patient was discharged a week after the application of the abdominal binder on midodrine 20 mg three times a day and his long-standing anticoagulant and anti-depressant. On midodrine alone with the abdominal binder inflated to 50 mmHg, his orthostatic vital signs were a blood pressure of 121/81 mmHg lying down with a heart rate of 89 bpm and blood pressure of 115/86 standing with a heart rate of 103 bpm. He continues to use the abdominal binder on a daily basis with ambulation without symptoms.

## Discussion

OH represents the inability to compensate for gravitational challenges on the heart and vascular system. With standing, approximately 500 to 1000 mL of blood pools in the splanchnic circulation and lower extremities leading to a decrease in the blood return to the heart, a reduction in cardiac output, and ultimately, blood pressure. To prevent this fall in pressure, the baroreceptor reflex is activated by the decline in pressure leading to increased sympathetic activity, increased peripheral vascular resistance, and increased chronotropy. This compensatory mechanism can be affected by a variety of conditions leading to OH, defined as a sustained drop in systolic blood pressure by ≤20 mmHg or diastolic pressure by ≤10 mmHg.

**Figure 2:**
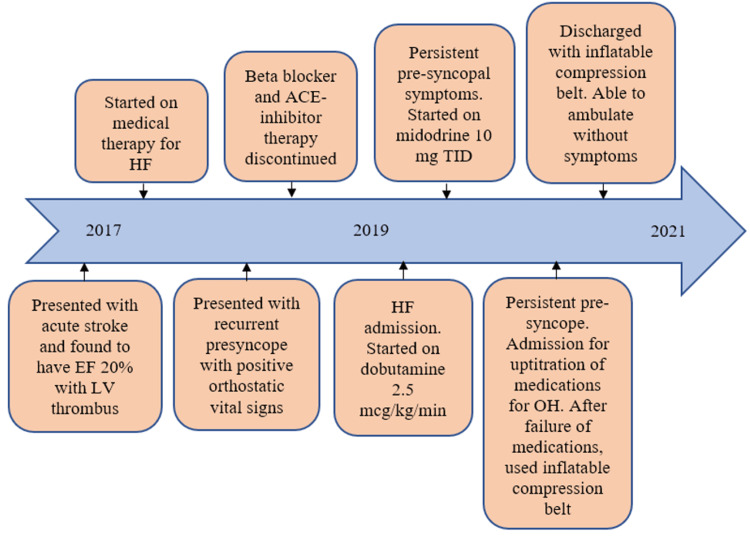
Clinical course The patient presented with an acute stroke in 2017 and was found to have a depressed ejection fraction with a mural thrombus. The progression of his symptom burden and orthostatic hypotension is depicted in this figure. HF - heart failure, EF - ejection fraction, LV - left ventricle, ACE - angiotensin-converting enzyme, TID - three times daily, OH - orthostatic hypotension Original figure created by authors

This case illustrates an extreme of OH in a patient with HF that was treated with a mechanical compression device (Figure 2). Studies have shown that patients with HF have a blunted change in blood pressure and heart rate due to increased venous pressure and volume and systemic adaption to low cardiac output [3]. Impaired splanchnic vascular capacitance in the human blood storage pool is felt to be one of the main protective mechanisms against OH in patients with HF. Patients with HF have an underlying increase in blood volume along with increased plasma renin and norepinephrine activity that helps protect against OH. Advanced forms of HF can lead to OH, primarily due to poor cardiac output. In the present case, the patient has developed autonomic failure with reduced cardiac filling pressures, eliminating the need for inotropic support. Diagnosis and treatment of OH in HF are critical to preventing morbidity and mortality while also helping to guide appropriate treatment options and advanced therapies. These patients can be particularly challenging from a volume management perspective as even patients with obvious signs of congestion can have OH [1]. Initial therapy should focus on reducing diuretics in patients with signs of intravascular volume depletion. Although HF goal-directed medical therapies can result in a decrease in blood pressure, they have been proven to improve cardiac function and have clear morbidity and mortality benefit. Clinicians should make every effort to continue HF therapies unless careful analysis suggests the risks significantly outweigh the benefits of treatment (Figure 1).

In patients with OH, the splanchnic vascular compartment is a well-recognized but often-underutilized therapeutic target for OH [4, 5]. Initial use of compression garments in the military space was reported in the 1940s, and in clinical space was in the 1960s. While abdominal binders provide a mild increase in blood pressure with standing, they are often difficult to use and not tight enough to provide adequate support. Inflatable systems like the belt shown in Figure 1 or a G-suit provide adjustable compression to the abdomen and lower extremities to counteract significant acceleration forces experienced by astronauts and pilots. Unfortunately, these suits are difficult to use for day-to-day activities in normal patients. Devices that apply adequate abdominal compression and/or lower leg compression to shift blood volume offer treatment for both neurogenic OH and OH in general (Figure 1). By focusing on abdominal compression, this allows for inflation to the minimum pressure to support the patient's blood pressure without leading to difficulty with circulation disturbance, as that could be a potential issue with lower leg compression.

## Conclusions

OH is a common medical condition that affects patients of all ages and comorbidities. Treatment is focused on intravascular volume expansion, medical therapy to raise blood pressure, and mechanical compression. OH is much less common and represents a particularly challenging phenotype in HF, given the conflicting goals of the treatment for OH and HF. Inflatable abdominal compression offers an effective treatment option for OH in patients with and without HF that should be further studied and utilized in the future.
